# The Effect of Age on Sentence Recognition in Noise with Different Noises Across the Adult Lifespan

**DOI:** 10.3390/audiolres16010025

**Published:** 2026-02-14

**Authors:** Ritik Roushan, Mohan Kumar Kalaiah, Usha Shastri, Kaushlendra Kumar, Gagan Bajaj, Megha M. Nayak

**Affiliations:** 1 Department of Audiology and Speech Language Pathology, Kasturba Medical College Mangalore, Manipal Academy of Higher Education, Manipal 576104, India; ritik.kmcmlr2022@learner.manipal.edu (R.R.);; 2 Department of Physiotherapy, Kasturba Medical College Mangalore, Manipal Academy of Higher Education, Manipal 576104, India

**Keywords:** sentence recognition, aging, noise, normal hearing

## Abstract

**Background/Objectives**: The present study examined the effect of age on sentence recognition in noise in different noise conditions among adults with normal hearing sensitivity throughout the adult lifespan. **Methods**: A total of 113 adults aged between 21 and 65 years participated in the study; based on age, they were categorized into five groups. The sentence recognition was assessed in five noise conditions: speech-shaped noise (SSN), amplitude-modulated speech-shaped noise (AM-SSN), two-male-talker babble (2MB), four-male-talker babble (4MB), and four-female-talker babble (4FB). The sentences were presented at a signal-to-noise ratio of −5 dB in all noise conditions. **Results**: The sentence recognition scores declined with increasing age in all noise conditions. In addition, age had a differential effect on the sentence recognition scores in the AM-SSN and 2MB conditions compared with the SSN, 4MB, and 4FB conditions. In the AM-SSN and 2MB conditions, the scores were significantly different in the fourth decade compared with young adults. In other noises, the scores were significantly different after 30 years compared with younger adults. Further, across noise conditions, greater scores were obtained in the AM-SSN and 2MB conditions, and the lowest scores were obtained in the 4FB condition. Partial Spearman correlations revealed a moderate-to-strong negative correlation between age and sentence recognition scores across noise conditions. **Conclusions**: The findings of the present study showed that sentence recognition is negatively affected by age. In addition, age has a differential effect on sentence recognition in different noises.

## 1. Introduction

Speech perception in noise is a complex auditory task that is essential for effective communication [1,2]. It plays an important role in daily life, since communication most often takes place in the presence of competing speech or noise. The process of speech perception begins with the auditory signal being received and processed by the peripheral auditory system, followed by the extraction of acoustic and phonetic details necessary for identifying speech sounds [3]. Several factors are known to affect the perception of speech-in-noise, such as the type of noise, signal-to-noise ratio (SNR), intensity of speech, age, hearing sensitivity, and auditory and cognitive processing abilities [4,5,6,7].

Aging is known to have a negative effect on the perception of speech in quiet and adverse listening conditions [8,9,10,11,12,13,14,15]. With advancing age, adults experience increasingly greater difficulty in understanding speech, especially in the presence of noise and reverberation. Several studies have investigated the effect of age on the perception of speech in quiet and adverse listening conditions using sentences [7,9,10,11,12,13,14,15], words [12,16,17], and non-sense syllables [12,18,19,20]. Most investigations comparing the speech perception abilities of young and elderly adults in quiet and noisy conditions have reported poorer speech perception in elderly adults [7,9,10,11,12,13,14,15,21,22,23]. Similarly, many studies comparing the performance of young and middle-aged adults have reported poorer speech perception in noise among middle-aged adults [9,10,13,14,16,20,21]. This decline in speech perception in quiet and noise with increasing age is attributed to age-related degenerative changes in the auditory system and cognitive abilities.

Noise affects speech perception by masking the acoustic features of the speech signal necessary for the recognition of speech sounds. The masking effects are explained based on two primary mechanisms: energetic masking and informational masking [24]. Energetic masking occurs when the background noise overlaps with the speech signal in frequency and time, thereby reducing the audibility of the speech [25]. Steady-state maskers, such as white noise and speech-shaped noise (SSN), primarily affect speech perception by energetic masking [25]. In contrast, informational masking results from the difficulty in segregating the target speech from competing signals (a process known as auditory scene analysis), especially when the masker shares acoustic and linguistic features with the target speech [24,25]. Competing speech and multi-talker babble primarily affect speech perception through informational masking. Previous studies have demonstrated that the extent of speech perception deficits in noise depends on the characteristics of the background noise, such as spectral and temporal fluctuations and the number of talkers in the speech babble [7,9,11,18,19,23].

Studies investigating the effect of age on the perception of speech-in-noise have shown greater speech perception difficulties in older adults than in young adults [8,9,10,11,12,13,14,15]. However, the difference in performance between the groups was not similar across different noises [7,21,22,23,26]. Specifically, greater difficulties have been reported among older adults in the single-taker and two-talker masker scenarios than in steady-state noise [21], in two-talker babble than in four-talker babble [26], in two-talker babble and speech-shaped noise than in amplitude-modulated noise [7,23], and in same-gender babble [7,21,22,23,26]. Based on these findings, the onset of speech perception difficulties in noise may begin at different ages depending on the noise type. Very few studies have investigated sentence recognition in noise across the adult life span [9,10,13,14,15]. However, most of the above studies have compared speech recognition thresholds in noise [9,10,13,14,15]. One study [10] compared sentence recognition scores in four-talker babble across the adult lifespan, and the results showed a significant reduction in sentence recognition in the fourth decade [10]. Similarly, studies measuring speech recognition thresholds in noise have shown a significant decrease in scores between 30 and 50 years across noises [9,10,13,14,15]. Given that different noises place distinct challenges for the perception of speech-in-noise, the present study aimed to investigate the effect of age on the perception of speech-in-noise in different noise conditions. The objective was to investigate the effect of age on the sentence recognition in noise in different noise conditions.

## 2. Materials and Methods

### 2.1. Participants

**Figure 1:**
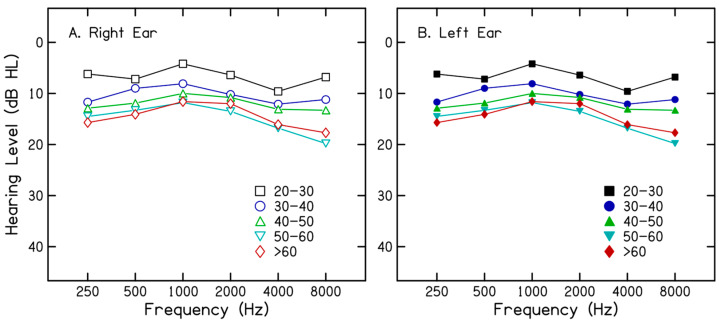
Mean hearing threshold (in dB HL) across frequencies and groups for the right ear (panel (**A**)) and left ear (panel (**B**)).

A total of 113 adults in five age groups participated in the study. The sample size was determined for a mixed design ANOVA (5 groups × 5 noise conditions) using G*power 3.1.9.7. To detect a medium effect size (f = 0.25) with a power of 0.80 and an alpha of 0.05, a minimum of 18 participants per group (*N* = 90) was required. Group 1 included 25 adults (male = 19; female = 6) aged between 21 and 30 years (mean = 24.4). Group 2 included 21 adults (male = 14; female = 7) aged 31–40 years (mean = 36.7). Group 3 included 23 adults (male = 12; female = 11) aged 41–50 years (mean = 45.8). Group 4 included 20 adults (male = 11; female = 9) aged 51–60 years (mean = 56.9). Finally, group 5 included 22 adults (male = 14; female = 8) aged 61–70 years (mean = 64.1). All participants had hearing sensitivity within normal limits in both ears with pure-tone thresholds less than 25 dB HL at octave frequencies from 250 Hz to 4000 Hz. Immittance evaluation showed type ‘A’ tympanograms and present acoustic reflex in both ears, suggesting no middle ear dysfunction. Further, none of the participants reported a history of otological problems such as ear pain; ear discharge; tinnitus; hearing loss; difficulty understanding speech in quiet, noise, or over the telephone; and exposure to hazardous noise or ototoxic medication. Figure 1 shows the mean hearing threshold across frequencies and groups for the right and left ears. In addition, all participants obtained a score of ≥25 on the Montreal Cognitive Assessment Test [27], indicating no evidence of cognitive impairment. The study was approved by the Institutional Ethics Committee, and all participants provided informed consent prior to their participation.

### 2.2. Stimuli

The sentence identification test in Kannada language, developed by Geetha et al. [28], was used for measuring sentence recognition ability. The test consists of 25 equivalent lists of sentences, with each list comprising 10 low-predictable sentences, each containing four keywords. The sentence recognition score was measured in five noise conditions. It included speech-spectrum-shaped noise (SSN), amplitude-modulated speech-spectrum-shaped noise (AM-SSN), two-male-talker babble (2MB), four-male-talker babble (4MB), and four-female-talker babble (4FB). The SSN and AM-SSN were generated using MATLAB (R2022b, MathWorks, Natick, MA, USA) at a sampling rate of 44,100 Hz with a 16 bit digital-to-analog conversion. To generate the SSN, all sentences were normalized to the same average root-mean-square (RMS) level, and an average spectrum was obtained. Using the averaged spectrum, a finite impulse response function was created, and white noise was passed through the impulse response function to obtain the SSN. The RMS amplitude of the SSN was adjusted with reference to the RMS amplitude of the sentences to obtain the desired signal-to-noise ratios (SNRs). The AM-SSN was generated by sinusoidally modulating the amplitude of the SSN at a rate of 4 Hz with a modulation depth of 90%.

**Figure 2:**
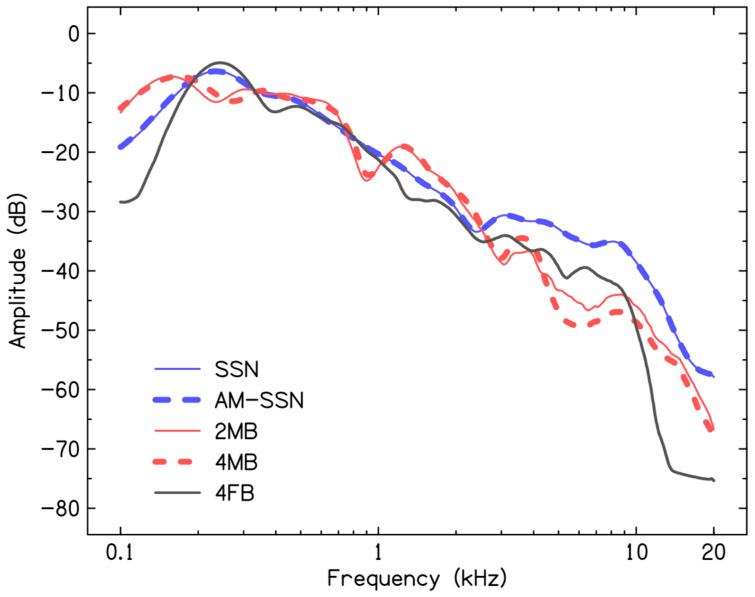
Long-term average spectrum of speech-shaped noise, amplitude-modulated speech-shaped noise, two-male-talker babble, four-male-talker babble, and four-female-talker babble used in the present study. It was obtained using the pwelch function in MATLAB, with a Hanning window of 20 ms duration with an overlap of 10 ms and a fast Fourier transform length of 2048 points.

The 2MB and 4MB were generated for the study, while the 4FB was available with the test material. To generating 2MB and 4MB, four males read a standardized Kannada passage [29] in the conversational style in a sound-treated room. The speech was recorded using a cardioid condenser microphone placed at a distance of 15 cm from the speaker’s mouth. The microphone was routed through an external sound card (Focusrite Scarlett Solo, Focusrite Audio Engineering Ltd., High Wycombe, United Kingdom) to ensure appropriate gain control. PRAAT software (version 6.0.39) was used to digitally record the speech at a sampling rate of 44,100 Hz with a 16 bit analog-to-digital conversion. To generate the babble noise, 5.5 s random speech segments from either two talkers (for the 2MB) or four talkers (for the 4MB) were extracted and digitally added using MATLAB. The 4FB was generated using a similar procedure, and the babble used in the present study was used in an earlier investigation [30]. The spectra of SSN, AM-SSN, 2MB, 4MB, and 4FB are shown in Figure 2.

### 2.3. Procedure

The sentence recognition in noise was measured in five noise conditions. In each noise condition, one list of sentences was presented to both ears at −5 dB SNR. The sentence lists were selected randomly for each noise condition to prevent order effects. In each trial, a new noise was generated and digitally mixed with the sentence. The noise always began one second prior to the onset of the sentence and ended one second after the offset. An auditory cue, consisting of a 1000 Hz tone with a duration of 250 ms, was provided immediately before the onset of the sentence to mark the beginning of the sentence. Participants were instructed to ignore the background noise and repeat the sentences. Prior to the formal testing, a practice trial was conducted in the SSN condition for all participants, with one list of sentences. After each trial, the number of keywords correctly identified in the sentence was noted. Following the participant’s response, the next sentence was automatically presented after an interval of 1.5 s. The mixture of sentence and noise was delivered binaurally using Sennheiser HD280 Pro headphones at the most comfortable loudness level of the participant.

### 2.4. Data Analysis

The total number of keywords correctly identified in each noise condition was computed for each participant and then converted to percent correct scores. Pure-tone thresholds from the right and left ears were averaged to obtain a single binaural PTA4 (500 Hz, 1000 Hz, 2000 Hz, and 4000 Hz) and pure-tone threshold at 8000 Hz for each participant. These variables were used as hearing-sensitivity measures for correlation analyses. To perform statistical analysis, the data were first examined for normality using the Shapiro–Wilk test. The results showed that the sentence recognition scores were not normally distributed in several groups (*p* < 0.05). Since the percent correct scores in all groups were not normally distributed, the percent scores were transformed to Rationalized Arcsine Units (RAUs). The transformed RAU scores were subjected to the Shapiro–Wilk test, but the results showed that the RAU scores were also not normally distributed in several groups. Thus, percent correct scores were considered for further statistical analysis. One-way ANOVA or the Kruskal–Wallis test was used to investigate the effect of age on sentence recognition scores in noise. The Friedman test was used to investigate the effect of the noise condition on the sentence recognition scores separately for each group. Partial Spearman’s rank correlation was performed to investigate the relationship between age and sentence recognition score with hearing sensitivity as a confounding factor. All statistical analyses were performed using JASP (Version 0.19.4) software.

## 3. Results

**Figure 3:**
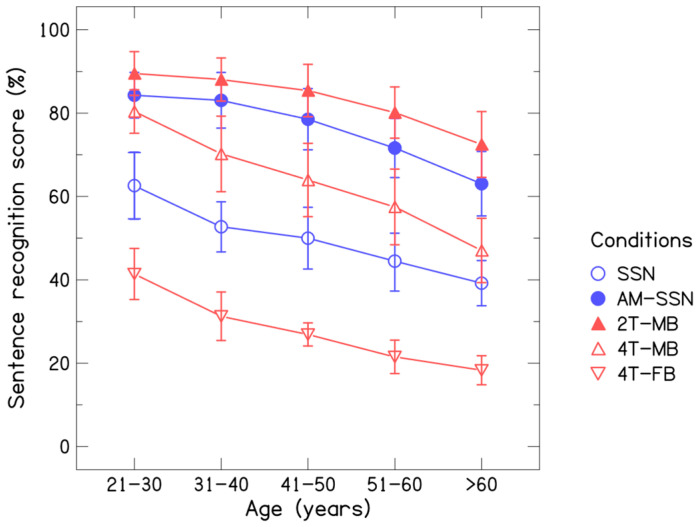
Sentence recognition score across noise conditions and age groups. Circles represent mean scores in speech-shaped noise (○) and amplitude-modulated speech-shaped noise (●), and triangles represent mean scores in two-male-talker babble (▲), four-male-talker babble (△), and four-female-talker babble (▽).

Table 1 shows the descriptive statistics for sentence recognition scores across age groups and noise conditions. The sentence recognition scores were highest in group 1 and declined systematically with increasing age in all noise conditions. Further, across noise conditions, the sentence recognition score was highest in the 2MB condition and lowest in the 4FB condition in all age groups. Figure 3 shows the performance trend for sentence recognition scores across age in different noise conditions.

### 3.1. Effect of Age on Sentence Recognition Scores Across Noise Conditions

To investigate the effect of age on sentence recognition scores, a one-way ANOVA was used in the SSN, AM-SSN, and 4MB noise conditions, and the Kruskal–Wallis test was used in the 2MB and 4FB conditions. The results showed a significant main effect of age on sentence recognition scores across all noise conditions (SSN: F(4, 108) = 38.85, *p* < 0.001; AM-SSN: F(4, 108) = 37.08, *p* < 0.001; 4MB: F(4, 108) = 57.11, *p* < 0.001, 2MB: H(4) = 54.7, *p* < 0.001; 4FB: H(4) = 87.83, *p* < 0.001). Pairwise comparisons were performed using the Bonferroni correction for the SSN, AM-SSN, and 4MB conditions. The results showed that, in both the SSN and 4MB conditions, scores were significantly different between all age groups, except between group 2 and group 3 (SSN: *p* = 1.000; 4MB: *p* = 0.094) and group 3 and group 4 (SSN: *p* = 0.068; 4MB: *p* = 0.091). Additionally, in the SSN condition, the scores were not significantly different between group 4 and group 5 (*p* = 0.194). Further, in the AM-SSN condition, scores were not significantly different between group 1 and group 2 (*p* = 1.000) and group 2 and group 3 (*p* = 0.540). Dunn’s post hoc comparisons were used for the 2MB and 4FB conditions. In the 2MB condition, scores were not significantly different between group 1 and group 2 (*p* = 0.430), group 1 and group 3 (*p* = 0.120), group 2 and group 3 (*p* = 0.430), group 3 and group 4 (*p* = 0.112), and group 4 and group 5 (*p* = 0.182). Finally, in the 4FB condition, the scores were not significantly different between group 2 and group 3 (*p* = 0.310) and between group 4 and group 5 (*p* = 0.310).

### 3.2. Effect of Noise Condition on Sentence Recognition Within Age Groups

To investigate the effect of noise condition on the sentence recognition scores, a Friedman test was performed separately for each group. The results showed a significant effect of condition on the sentence recognition scores across groups (group 1: χ^2^(4) = 90.83, *p* < 0.001; group 2: χ^2^(4) = 87.34, *p* < 0.001; group 3: χ^2^(4) = 91.77, *p* < 0.001; group 4: χ^2^(4) = 77.69, *p* < 0.001; group 5: χ^2^(4) = 85.5, *p* < 0.001). Conover’s post hoc comparison revealed that scores were significantly different between all conditions (*p* < 0.05) across groups.

### 3.3. Relationship Between Age and Sentence Recognition Score

**Figure 4:**
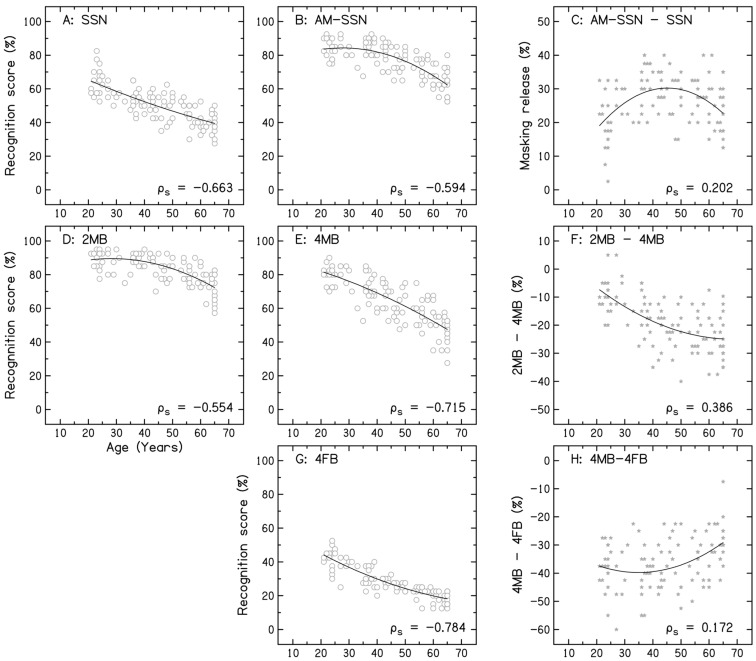
Scatterplot showing the relationship between age and sentence recognition score in speech-shaped noise (panel (**A**)), amplitude-modulated speech-shaped noise (panel (**B**)), two-male-talker babble (panel (**D**)), four-male-talker babble (panel (**E**)), and four-female-talker babble (panel (**G**)). Panel (**C**) shows the improvement in identification scores (masking release) for amplitude-modulated speech-shaped noise relative to the unmodulated condition. Panel (**F**) shows the difference in performance when the number of talkers was increased from two to four in male-talker babble. Panel (**H**) shows the difference in performance between the four-male-talker and four-female-talker babble conditions.

The scatterplots in Figure 4 (panels A, B, D, E, G) show the relationship between age and sentence recognition scores in various noise conditions, with the trendlines representing the polynomial fit. The scores in the AM-SSN and 2MB noise conditions showed a significant decline between 35 and 40 years of age. The trendline also shows an accelerated decline beginning in early middle age. In contrast, scores in the SSN, 4MB, and 4FB conditions showed an earlier decline beginning at 30 years of age. Panel C shows the release from masking (AM-SSN−SSN) for sentence recognition across age. The release from masking increased with age in young adults, peaked in middle-aged adults, and subsequently declined with age in older adults. Panel F shows the magnitude of reduction in sentence recognition scores across age as the number of talkers in the babble increased from two to four (2MB to 4MB). The magnitude of reduction in performance increased progressively with age, suggesting greater difficulty among middle-aged and older adults than among young adults. Panel H shows the magnitude of reduction in sentence recognition scores when the gender of the target speech talker was the same as that of the talkers in the babble. The reduction in performance was similar across the age range.

To investigate the relationship between age and sentence recognition scores, a partial Spearman’s rank correlation analysis was carried out separately for each noise condition, with binaural PTA4 and pure-tone threshold at 8 kHz as confounding variables. The results showed a significant negative correlation between age and sentence recognition scores across all noise conditions (SSN: ρ_s_(109) = −0.663, *p* < 0.001, 95% CI = [−0.748, −0.550], AM-SSN: ρ_s_(109) = −0.594, *p* < 0.001, 95% CI = [−0.698, −0.475], 2MB: ρ_s_(109) = −0.554, *p* < 0.001, 95% CI = [−0.641, −0.379], 4MB: ρ_s_(109) = −0.715, *p* < 0.001, 95% CI = [−0.793, −0.615], 4FB: ρ_s_(109) = −0.784, *p* < 0.001, 95% CI = [−0.860, −0.674]).

## 4. Discussion

The present study examined the effect of age on sentence recognition scores in noise across different noise conditions among adults with normal hearing sensitivity throughout the adult lifespan. The results showed an age-related decline in sentence recognition scores across all noise conditions. These results of the present study align with the findings of previous research in the literature [9,10,13,14,15]. Further, age had a differential effect on performance in the AM-SSN and 2MB conditions compared with the SSN, 4MB, and 4FB conditions. In AM-SSN and 2MB, sentence recognition ability was significantly poorer in middle-aged and older adults than in young adults. Further, a significant decline in performance was noted during early middle age between 35 and 40 years. In contrast, performance in the SSN, 4MB, and 4FB conditions began to decline significantly earlier, starting at 30 years of age. These are novel results found in the present study. Several studies have investigated the effect of age on speech perception, but few studies have explored the trajectory of age-related decline across the adult lifespan [9,10,13,14,15]. Among these studies, the majority investigated the effect of age on the speech recognition threshold in noise (SNR-50) [9,11,13,14,15,16], while one study reported correct recognition scores similar to those in the present investigation [10]. In comparison to the findings of the present study, a significant decline in SNR-50 in SSN was reported around 50 years of age in two investigations [11,13] and at 38 years in one investigation [14]. Similarly, a significant decline in SNR-50 in white noise was noted after 40 years of age [15]. Further, Lunardelo et al. [15] noted that the onset of decline in SNR-50 in white noise was around 30 years of age, but the decline was significant around age 40. Finally, Jain [10], in an unpublished study using Kannada sentences as in the present study with four-talker babble, found a significant decline in identification scores after 40 years of age. Thus, the findings of the present study are comparable to results documented in the literature. In SSN, the onset of decline in performance was 30 years, which is comparable to the findings of Lunardelo et al. [15] in white noise. In speech babble, a significant decline was noted between 35 and 40 years, which is also comparable to the findings of Jain [10].

The age-related decline in speech perception is well documented among older adults in both quiet and noisy listening conditions. Recently, several investigations have revealed that these changes begin early in middle age. Studies have reported poorer speech perception in middle-aged and older adults using consonant–vowel syllables, words, and sentences in quiet and noisy listening conditions [7,9,10,11,13,14,15,16,17,18,19,20]. The decline in speech perception with aging is generally attributed to hearing loss, poorer supra-threshold auditory processing, and cognitive decline associated with aging. In the present study, we included participants with normal hearing sensitivity up to 4000 Hz in both ears to overcome the effects of hearing loss on sentence recognition. In addition, hearing sensitivity was considered a covariate during the statistical analysis. Despite these controls, the correlation analysis showed a significant relationship between sentence recognition scores and age across noise conditions, which indicates that hearing sensitivity alone does not fully account for age-related changes in speech perception. In addition, age-related cognitive changes may have also contributed to the observed decline in sentence recognition scores. However, the effects of cognitive changes could not be investigated as the cognitive abilities of the participants were not measured.

To understand the interaction between age and noise characteristics, the study also investigated the effect of different noises on the sentence recognition scores. The results showed superior sentence recognition scores in the AM-SSN and 2MB conditions and lowest scores in the 4FB condition. These findings in the present study are consistent with previous investigations [7,11,18,21,22,23,26]. Many studies have reported superior speech perception in amplitude-modulated noise compared with steady-state noise [7,11,18,23]. This improvement is attributed to the glimpsing effect or listening in the dips. The temporal fluctuations in the AM-SSN and 2MB provide opportunities for listening in the dips, which facilitates the partial recovery of speech during momentary reductions in the level of masking noise [25,31]. The poorest score in the 4FB condition could be explained based on the voices of talkers in target sentences and babble. Studies have shown that, as the difference in F0 increases between the target speech and babble, speech perception scores also increase [24,26,32]. The improvement in speech perception is attributed to the ability to discriminate or segregate the voices in the target speech and babble [26,32]. Thus, the poorest performance in 4FB in the present study could be attributed to difficulty in segregating the target voice or sentence in the presence of babble. Further, between the 2MB and 4MB conditions, sentence recognition was poorer in the 4MB condition. This decline in intelligibility is attributed to the reduction in glimpsing opportunities when the number of talkers increases in the babble [26,33]. When the number of talkers increases in the babble, the competing speech overlaps in time and frequency and fills the spectral and temporal dips, thereby reducing the glimpsing opportunities. In addition, as the number of talkers increases, the babble acoustically resembles steady-state noise (or SSN) and increases energetic masking, leading to poorer speech perception [33].

Finally, the results of the present study showed that the release from masking in AM-SSN was reduced in older adults compared with middle-aged adults. Further, when the number of talkers was increased in the babble, the magnitude of reduction in the performance increased progressively with age. A similar disproportionate decline in performance has been reported in older adults when the number of talkers was increased in the babble [19,21]. These findings could be a consequence of age-related deficits in temporal processing abilities [21,31]. Many studies have reported poorer auditory temporal resolution, temporal envelope, and fine-structure processing in older adults, which is considered important for listening in dips and segregating auditory streams [21,31,34,35].

One of the limitations of the present study is that sentence recognition was measured only at one SNR (−5 dB). Utilizing multiple SNRs would have provided a more comprehensive understanding of the effect of age across varying levels of difficulty. Thus, the findings of the present study may not be generalized to other SNRs. Another limitation is that, in the female-talker babble condition, only 4FB alone was used, and sentence recognition in 2FB was not measured. Thus, comparison between 2FB and 4FB is not possible. Third, the cognitive abilities of participants were not measured; thus, confounding effects of these abilities on the relationship between age and sentence recognition were not considered. Future studies should incorporate multiple SNRs and measures of cognitive function to understand the effects of age on speech perception difficulties across adulthood.

## 5. Conclusions

The findings of the present study showed an age-related decline in sentence recognition scores across all noise conditions. In addition, age appeared to have a differential effect on performance in highly fluctuating noises and in steady or less-fluctuating noises. The decline in sentence recognition scores in highly fluctuating noises was noted at a later age than that in steady or less fluctuating noises.

## Figures and Tables

**Table 1:** Mean and standard deviation (in parenthesis) and median and interquartile range (in parenthesis) for sentence recognition scores across groups and noise conditions.

	SSN		AM-SSN		2MB		4MB		4FB	
Group	Mean (SD)	Median (IQR)	Mean (SD)	Median (IQR)	Mean (SD)	Median (IQR)	Mean (SD)	Median (IQR)	Mean (SD)	Median (IQR)
Group 1	62.6 (8.1)	60.0 (10.0)	84.3 (5.5)	85.0 (10.0)	89.5 (5.2)	92.5 (7.5)	80.4 (5.2)	80.0 (5.0)	41.4 (6.1)	42.5 (7.5)
Group 2	52.7 (6.0)	52.5 (5.0)	83.1 (6.7)	85.0 (7.5)	88.1 (5.2)	90.0 (6.9)	70.2 (9.1)	70.0 (13.1)	31.2 (5.8)	30.0 (10.0)
Group 3	50.0 (7.4)	51.3 (12.5)	78.5 (7.3)	80.0 (12.5)	85.4 (6.3)	86.3 (8.1)	63.9 (8.8)	66.3 (13.1)	26.9 (2.8)	27.5 (3.1)
Group 4	44.2 (6.9)	43.8 (10.0)	71.6 (7.1)	71.3 (10.6)	80.1 (6.1)	78.8 (10.0)	57.5 (9.1)	57.5 (13.1)	21.5 (4.0)	22.5 (3.1)
Group 5	39.2 (5.4)	40.0 (4.3)	63.1 (7.7)	62.5 (12.5)	72.5 (7.9)	75.0 (14.4)	47.1 (7.7)	48.8 (9.4)	18.3 (3.5)	18.8 (6.9)

Note: SD, standard deviation; IQR, interquartile range; SSN, speech-shaped noise; AM-SSN, amplitude-modulated speech-shaped noise; 2MB, 2-male-talker babble; 4MB, 4-male-talker babble; 4FB, 4-female-talker babble; scores in percentage (%).

## Data Availability

The raw data supporting the conclusions of this article will be made available by the authors on request.

## Abbreviations

The following abbreviations are used in this manuscript:

SNR	Signal-to-noise ratio
SSN	Speech-shaped noise
AM-SSN	Amplitude-modulated speech-shaped noise
2MB	Two-male-talker babble
4MB	Four-male-talker babble
4FB	Four-female-talker babble
RMS	Root mean square
PTA	Pure-tone average
RAU	Rationalized arcsine unit
